# Deep Reinforcement Learning-Assisted Optimization for Resource Allocation in Downlink OFDMA Cooperative Systems

**DOI:** 10.3390/e25030413

**Published:** 2023-02-24

**Authors:** Mulugeta Kassaw Tefera, Shengbing Zhang, Zengwang Jin

**Affiliations:** School of Cybersecurity, Northwestern Polytechnical University, Xi’an 710072, China

**Keywords:** deep reinforcement learning, distributed optimization, game theory, power control, throughput maximization, wireless interference channel

## Abstract

This paper considers a downlink resource-allocation problem in distributed interference orthogonal frequency-division multiple access (OFDMA) systems under maximal power constraints. As the upcoming fifth-generation (5G) wireless networks are increasingly complex and heterogeneous, it is challenging for resource allocation tasks to optimize the system performance metrics and guarantee user service requests simultaneously. Because of the non-convex optimization problems, using existing approaches to find the optimal resource allocation is computationally expensive. Recently, model-free reinforcement learning (RL) techniques have become alternative approaches in wireless networks to solve non-convex and NP-hard optimization problems. In this paper, we study a deep Q-learning (DQL)-based approach to address the optimization of transmit power control for users in multi-cell interference networks. In particular, we have applied a DQL algorithm for resource allocation to maximize the overall system throughput subject to the maximum power and SINR constraints in a flat frequency channel. We first formulate the optimization problem as a non-cooperative game model, where the multiple BSs compete for spectral efficiencies by improving their achievable utility functions while ensuring the quality of service (QoS) requirements to the corresponding receivers. Then, we develop a DRL-based resource allocation model to maximize the system throughput while satisfying the power and spectral efficiency requirements. In this setting, we define the state-action spaces and the reward function to explore the possible actions and learning outcomes. The numerical simulations demonstrate that the proposed DQL-based scheme outperforms the traditional model-based solution.

## 1. Introduction

Interference management for multi-cell networks has recently attracted increasing concerns from physical layer design and resource allocation. The large deployment of base stations (BSs) overlaying the coverage area of point-to-point wireless connections results in a multi-cell interference system [1]. OFDMA, by its nature, is immune to intra-cell interference due to the allocation of orthogonal sub-carriers to the corresponding users. However, inter-cell interference becomes a severe threat to users due to the joint transmission strategies across multiple cells. It becomes more compounded for edge users where multiple BSs are deployed in conjunction with neighboring transmitter-receiver pairs. For effective inner-cell interference (ICI) mitigation, cooperative transmission strategies have been investigated extensively in [2]. The source transmitter may increase the power transmission to maximize its spectral efficiency, but it may degrade the channel quality of neighboring transmitter-receiver pairs [3]. With such a technique, the cross-coupling transmission of BSs occupying the same time slot and resource allocation can simultaneously send their respective information. Consequently, the adverse impact of ICI on its neighbors’ transmissions will reduce gradually.

In multi-cell networks, network densification and optimal resource allocation will result in new challenges for the design and optimization of the entire system. Traditionally, numerical optimization is the dominant approach to resource allocation problems [4]. The commonly used optimization algorithms include weighted-minimum mean squared error (WMMSE) [5], fractional programming [6], and interference pricing [7]. These algorithms typically adopt iterative approaches that take crucial performance indicators such as channel realization as input and output the results as an optimal power allocation strategy. Despite the remarkable success of these algorithms, most of the optimization problems are hard to solve due to their non-convex nature and high-dimensional optimization parameters [8]. Moreover, the increasing heterogeneity of the upcoming 5G networks, which combine a variety of new features with more complex communication requirements, will intensify the resource allocation problems, making the conventional model-based approaches hard to solve mathematically [9]. Despite the computational complexity and lack of precise models, these algorithms must be reformulated again since the CSI is time-varying, and resource management may only be feasible for a short practical success. The future communication system will require dynamic, robust algorithms to adapt network architectures and resource management for different services in diverse scenarios. Therefore, motivated by the departure from traditional design concepts, a more flexible method for wireless resource allocation is required to react to new situations. This underlying process can be achieved based on learning new features and their consequences on system performance metrics [10].

The development of machine learning (ML) techniques, especially the deep learning (DL) method, improve the quality of resource allocation by learning efficient representations of data from unstructured sources rather than pre-established massive data sets [11]. It provides a powerful data-driven method to improve resource allocation problems by giving a more efficient allocation and scheduling system. Reinforcement learning (RL) concerns how intelligent agents must perform specific actions to maximize the expected rewards to achieve their ultimate goal. In particular, RL shows a promising approach, where the agents interact with the wireless environment, and aggregated learning happens over the environment. The objective of RL is to find an optimal strategy that maximizes the specific outputs (training) from any given state in the observed environment. The work in [12] studies the critical motivations of using RL for allocating and managing wireless resources with application to vehicular networks. More specifically, it has been shown that the deep reinforcement learning (DRL) method can provide a viable solution that is hard to model and solve precisely in the traditional framework. Furthermore, many studies have been directed related to the application of DRL in 5G network optimizations [13,14,15]. With all these discussions above, DRL for resource management in wireless networks remains immature, owing to the difficulty in traditional design concepts uniquely modeling the behavior of underlying processes subject to input and producing the optimum resource allocation strategy. Hence, there is a need for further enhancing the DRL techniques to ensure efficient resource allocation and user service requirements in a distributive optimization manner.

In this paper, we consider distributed interference management for the downlink OFDMA system. Each BS collects CSI and QoS information from neighboring transmitter-receiver pairs and uses its own transmit power accordingly. In particular, we first formulate the corresponding power allocation as a non-cooperative game model, where BSs compete for resource allocations by maximizing their utility data rates. Then, we develop a DQL model to be employed by all BSs to learn their optimal power allocation strategy in multiple interference channels. The ultimate goal is to maximize the system’s overall sum rate while ensuring each user’s QoS constraints. The main challenges in this paper are listed as follows: (1) since the considered OFDMA system has multiple cells and mobile terminals, there are inter-cell and intra-cell co-channel interferences, making the RB allocation more complicated and challenging than a single-cell system. (2) the optimization problem is NP-hard combinatorial problem with nonlinear constraints. How to efficiently solve this non-convex and NP-hard combinatorial optimization problem is a nontrivial task. (3) since we also consider the throughput evaluation of each user with the target of maximizing the overall sum rate of the network, it may be challenging to achieve optimal power allocation when the required throughput of the user is too large. In this case, it is challenging to solve whether an optimal solution satisfies the throughput requirements as the size of the systems increases. To address these challenges mentioned above, we utilize DQN with a QoS requirement threshold for each user in a multi-cell network. The DQN estimates the relationship between available CSI and the solution of the optimization problem. A DQN agent is then used to compute the joint power control policy and user scheduling across the multi-cells to adapt the channel condition of the entire network. The important contributions of this paper are outlined as follows:

▪ We first formulate the optimization problems as a non-cooperative game model, where each BSs is considered a game player. In particular, the resource allocation problem of a distributed interference network under non-linear constraints has been solved using the Nash equilibrium solution concepts.
▪ Then, a downlink power allocation scheme based on DRL has been presented to reduce computational complexity and make the solution feasible. We define the state-action spaces and reward signal design for the DRL agent for evaluating the possible actions and learning outcomes.
▪ Since the DRL-based solution is model-free, it does not depend on the network size and a large amount of training data. Unlike the supervised learning technique, there is no need to execute an optimal strategy to design a massive training dataset. This makes the proposed DRL-based optimization scheme less complex and scalable for large-scale systems.
▪ We provide numerical simulations to assess the performance of the proposed scheme. Simulation results indicate that the proposed DQL approach significantly improves the optimization of transmit strategies.

The remainder of this paper is structured as follows. A review of related works is presented in Section 2. Section 3 introduces the system model and problem formulation using a non-cooperative game model. In Section 4, we present our proposed DQL-based resource allocation approach in detail. Section 5 provides the numerical simulations and discussions, followed by a conclusion in Section 6.

## 2. Related Work

Resource allocation problems have been extensively studied in the literature for multi-cell wireless systems [2,16,17,18]. For this setting, optimally allocating the power control and user scheduling is essential to improve the achievable sum-rate performance [19,20]. While most of these studies use iterative approaches to maximize the overall system throughput, the optimization problems are non-convex due to the nonlinear probabilistic constraints [21]. One of the alternative approaches to deal with non-convex optimization problems is through the use of a game theoretical model. These techniques allow each cell to independently optimize its transmission power in a non-cooperative game model [22,23,24]. Despite the remarkable success of this optimization model, it is also known that a non-cooperative game solution is often practically inefficient in the case that either the user’s QoS requirements are not satisfied, or the performance of the channel condition is poor [25]. In such situations, multiple-cell cooperation can improve system performance by allowing user data to be jointly processed by several interfering transmitters (BSs). Along with many advantages, such as higher spatial degrees of freedom, expanded cell coverage, improved signal quality at destination receivers, etc., comes with cross-cell interference and computational complexity, which inevitably results in the reduction of entire network performance.

Deep reinforcement learning (DRL) techniques have recently been applied to solve various resource management problems, including spectrum access, throughput maximization, power, and channel allocation, which all enhance the 5G wireless networks [26,27,28]. In [29], the classical Q-learning technique was employed for downlink resource allocation in non-orthogonal multiple access (NOMA) networks. The classical Q-learning algorithm uses a lookup table to store all the action-value functions. Though classical Q-learning can help to handle the complexity of traditional model-based solutions, it will need to be able to model the design criteria exactly, especially for high dimensional state spaces [30]. More specifically, it is hard to store all the values of state-action pairs in a tabular form when the state space of the problem is too large.

To deal with the aforementioned challenges, the DRL technique has been considered. On the other hand, purely based on the training over DQN with experience replay, DQL can help handle the problems that could not be solved in the traditional learning system. In [31], a DQL was used for power allocation in a cloud-RAN to reduce power consumption while ensuring the reliability constraint of each user. A distributed DQL-based spectrum-sharing scheme was proposed in [32] for multiple users in a non-cooperative manner. In [33], a DRL-based method was proposed to address the heterogeneous network’s joint user association and resource allocation (UARA). The goal in [33] is to maximize the future utility functions of the overall system while ensuring the QoS constraints in a heterogeneous downlink system. The DRL-based resource allocation approach to maximize the overall sum throughput in multi-user cellular networks has also been considered [34]. Ref. [35] uses a multi-agent DQL model to determine the dynamic and optimal power allocation in wireless networks. A DQN function was used to solve the instability problem of the classical Q-learning technique. The ultimate goal is to increase the weighted sum throughput of the system in a distributed fashion. In [36], a deep Q-learning learning (DQL) approach was proposed for centralized power allocation to improve the total throughput of the network in multiple cell systems. Furthermore, a DRL with the link outage constraint was developed in [37] to minimize the complexity of the resource allocation issue in a wireless interference system. Specifically, the authors utilized a DQL approach to remove the inherent instability in the traditional learning process.

DRL can also be applied to different function blocks in communication networks, such as end-to-end design, slice management [38], mobile edge computing [39], etc. In [40], the authors construct a DNN-based end-to-end system optimization model to reduce the data at the transmitter end while improving the decoding accuracy. For the joint optimization of different blocks, the DL approach can utilize a data-driven model based on expert knowledge and a big data system [41,42]. Furthermore, the authors [43,44] provide model-based optimization approaches in the physical layer. DL can also be integrated with different iterative estimation approaches to train the required DRL parameters and full automation of the system [45]. However, optimizing DRL parameters is a key issue in evaluating the agent’s final performance metrics [46]. Bayesian optimization [47] has recently achieved significant success in solving a hyperparameter optimization problem. Furthermore, the neural architecture assistant search framework was proposed in [48,49] for optimizing hyperparameters and predicting the accuracy of the final learning outcome.

The work in [25] uses the generalized Nash equilibrium problem (GNEP) algorithm to optimize transmitted power control while assigning the same RB in multiple cells. The formulated GNEP analyzes the optimization problem of small cell networks via variational inequality theory. Lagrange multipliers are proposed to evaluate the actions of the Q function while ensuring global QoS requirements. The proposed approach in this paper uses both the game theoretical formulation and the DQL algorithm to address the optimization of downlink power allocation in multi-cell networks. In particular, the DQL-based distributive algorithm is used to evaluate the actions of the Q function while avoiding the same RB allocation to users in neighboring cells that are located close to the cell edge. Because, in such situations, the users involved in the neighboring transmitter-receiver pairs cannot obtain essential SIR to work correctly regardless of the transmitting power of interfering BSs.

## 3. Problem Statement and Formulation

### 3.1. System Model 

**Figure 1:**
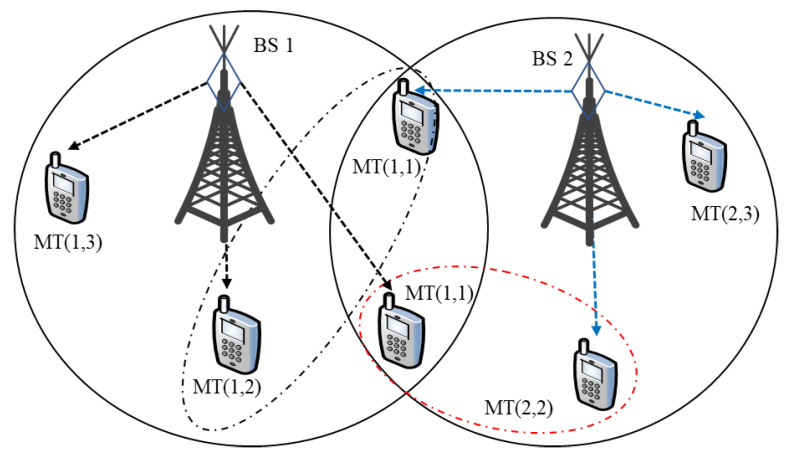
An illustration of downlink resource allocation for multi-cell and multiple user systems.

We consider a downlink OFDMA system, as depicted in Figure 1, where multiple transmitters (BSs) communicate with multiple users within each cell and share one frequency band per cell. The multiple BSs share the downlink resource with each user and spread a resource block (RB) in OFDMA. We assume that the transmit and receive terminals are equipped with a single antenna. Note that the OFDMA RBs allocated to different users within the same cell are orthogonal to each other since the intra-cell interference does not exist. Hence, the inter-cell interference across different cells is the leading performance limiting factor for throughput evaluation.

For convenience, we denote the transmitters as BS and the set of active users as $U_{i},$ i=1,2,…,K that employed with sub-carriers in multi-cell networks. The direct channel gain between the $i^{th}$ BS-user pair over channel n is denoted by $h_{i,i}^{n}$ and the interference channel between the $j^{th}$ BS and the $i^{th}$ user over channel n is denoted by $h_{i,j}^{n}$. Therefore, the received signal at $i^{th}$ user over channel n is given by

(1) $$Y_{i}\left(n\right)=h_{ii}^{n}x_{i}+\sum_{l\neq k}^{K}h_{ij}^{n}x_{j}+n_{i}$$

Here, $n_{i}\;$ is the AWGN power spectral density (PSD) at destination user i with distribution $n_{i}\sim N\left(0,\sigma_{i}{}^{2}\right)$ such that $\sigma_{i}>0$. Note that the confidential messages transmitted by different sources are independent of each other. In such cases, each source message can be kept confidential from all other unintended users. The transmitting power of a BS i in channel n is represented as $P_{i}^{n}$. We also represent the downlink power allocation vector of the system as $p≜vec\left\{p^{\left(1\right)},\ldots ,p^{\left(N\right)}\right\}$, where $p^{\left(n\right)}≜{\left(P_{1}^{n},\ldots ,P_{i}^{n}\right)}^{T}$. Therefore, the SINR for the $i^{th}$ user over channel n can be written as

(2) $$SINR_{i}=\frac{P_{i}^{n}{\left|h_{ii}^{n}\right|}^{2}}{\sum_{j\neq k}^{K}P_{j}^{n}{\left|h_{ij}^{n}\right|}^{2}+\sigma_{i}{}^{2}}$$

Accordingly, the corresponding achievable rate at the $i^{th}$ user served by BS i under channel n can be expressed as

(3) $$R_{i}=\log_{2}\left(1+SINR_{i}\right)$$

The achievable data rate $R_{i}$ can be maximized using the optimization of transmit power constraints. It is to be noted that the power of AWGN at each destination user has been normalized, while the effects of multipath fading and path loss have been modeled as a location-dependent channel variance.

### 3.2. Problem Formulation

In this paper, our target of optimizing the power allocation is to maximize the overall sum rate of all wireless channels under the important constraints, such as power and spectral capacity requirements. We first formulate the objective function as a Gaussian interference game (GIG) under transmit power constraint by considering the discrete frequencies [50]. For simplicity, we assume an increasing sequence of frequencies such that $f_{o}<\cdots <f_{k}$. In particular, we adopt a discrete approximation game to model the GIG from $j^{th}$ BS to $i^{th}$ receiver over K frequency channel as $GI_{\left\{I_{1,\;\ldots ,I_{k}}\right\}}$, where $I_{k}$ is the closed interval given by $I_{k}=\left\{f_{k-1},f_{k}\right\}$. Specifically, each BS is considered as a game player, i.e., there are K parallel frequency channels to operate as 1,…,N over K. The player can send a power vector $P_{i}=\left(P_{i}(1\right),\;\ldots ,\;P_{i}\left(k\right))\in {\left[0,P_{i}\right]}^{k}$, such that $P_{i}\left(k\right)$ is the transmit power in the closed interval $I_{k}$. Hence, we will have $\sum_{k=1}^{K}P_{i}\left(k\right)=P_{i}$. This indicates that the set of power allocations for all receivers is a closed convex subset of the cube $\prod_{i=1}^{N}{\left[0,P_{i}\right]}^{k}$, denoted by

(4) $$B=\prod_{i=1}^{N}B_{i}$$

where $B_{i}$ denotes the set of power allocations for player i. Mathematically, $B_{i}$ can be expressed as

(5) $$B_{i}={\left[0,P_{i}\right]}^{k}\left\{\left(P_{i}(1\right),\;\ldots ,\;P_{i}\left(k\right)):\sum_{k=1}^{K}P_{i}\left(k\right)=P_{i}\right\}$$

Here, each player selects a PSD $P_{i}=P_{i}\left(k\right):1\leq k\leq N\in B_{i}$. The spectral capacity of each player i is its sum rate $R_{i}$, and each player has to satisfy the power and QoS requirements. Note that the QoS constraint depends on the BS’s transmitting power, indicating that it shall be optimized to achieve the target of service request. Accordingly, the optimization game is formulated as:

(6) $$\underset{p>0}{\max}\sum_{i=1}^{K}w_{i}R_{i}\left(k\right)\;s.t.\;0\leq P_{i}\left(k\right)\leq P_{i}^{max}\;SINR_{i}\left(k\right)>\gamma_{o}\left(k\right),\;k=1,\ldots ,K$$

where $w_{i}$ is the given non-negative weight of the sub-channel assignment at the $i^{th}$ BS to maximize the total sum throughput. Here, we assume $w_{i}=1$ for each player i while $P_{i}^{max}$ is the maximum power the BS can use it. The objective of Problem (6) is to maximize the achievable rate at the destination user so that they can obtain useful information with the desired service request, which is always defined by the constraint of the SINR higher than or equal to the given threshold value of the $i^{th}$ user, i.e., $SINR_{i}\left(k\right)>\gamma_{o}\left(k\right),\;k=1,2,\ldots ,\;K$.

In the literature, the formulation problem in (6) has been shown to be an NP-hard combinatorial problem due to the nonlinear probabilistic constraints [8]. No convex reformulation of the above problem is known, even without the QoS constraints. Therefore, the directly optimal solution is non-trivial and may not be feasible. In the following, we will show that the above problem in (6) can be unified under the Gaussian interference game (GIG) framework to simplify the formulation. Specifically, we will first analyze the payoff for each user i and provide a payoff vector R for finding the GIG of Problem (6). Then, we will describe the Nash equilibrium solution to achieve a stationary solution of the given power allocation p.

For ease of exposition, let us consider the transmit power distributions for player i, such that each player selects a power $P_{i}=P_{i}\left(k\right):1\leq k\leq N\in B_{i}$. Thus, the utility rate for user i is the downlink spectral efficiency of link i, which is given by:

(7) $$R^{i}\left(p_{1},\ldots ,p_{N}\right)=\sum_{k=1}^{K}\log_{2}\left(1+SINR_{i}\left(k\right)\right)\Delta f_{k}$$

where

(8) $$SINR_{i}\left(k\right)=\frac{P_{i}\left(k\right){\left|h_{ii}\left(k\right)\right|}^{2}}{\sum_{j\neq k}^{K}P_{j}\left(k\right){\left|h_{ij}\left(k\right)\right|}^{2}+\sigma_{i}{}^{2}\left(k\right)}$$

$R^{i}$ is the available utility function to player i with given power allocations $p_{1},\ldots ,p_{N}$ while $\Delta f_{k}$ is the bandwidth of the $k^{th}$ interval. As defined above, $h_{ii}\left(k\right)$ and $h_{ij}\left(k\right)$ are the direct channel gains and cross coupling functions, respectively. Here, we consider two special cases for the noise term in (8). In the first case, when the noise term $\sigma_{i}{}^{2}\left(k\right)>0$, there is an external noise in the $i^{th}$ user at frequency k and optimization problem becomes more complex. In the second case, when $\sigma_{i}{}^{2}\left(k\right)=0$, the noise term can be ignored, and the spectral efficiencies might become large using FDM strategies. Hence, the available capacity $R^{i}$ for each player i is continuous over multiple channels. 

Our goal is to model the payoff vector R for frequency response, so we can simplify the problems for N-player games. Let the GIG is given by $GI_{\left\{I_{1,\;\ldots ,I_{k}}\right\}}=\left\{R,\;B\right\}$ for N-players, the continuous payoff vector R be denoted as

(9) $$R=\left\{R^{1},\ldots ,R^{N}\right\}$$

where B and $R^{i}$ are the strategy set and the available capacity defined in (4) and (7), respectively. Moreover, we also consider a Nash equilibrium (NUM) problem intending to optimize the entire network performance under the constraints power budget and QoS conditions. Note that the QoS requirements are applicable if and only if the game players are satisfied without cross-coupling interference from the interfering transmitters. Given all other players i and player n in a p strategy, the Nash equilibrium for QoS constraints is formulated as [51]

(10) $$R^{i}\left(p_{1},\ldots ,p_{n-1},\;p,\;p_{n+1},\ldots ,p_{N}\right)\leq R^{n}\left(p_{1},\ldots ,p_{N}\right)$$

It can be seen that the problems mentioned above are homogeneous with respect to power allocation and QoS requirements, thereby depending on the power and QoS constraints. Specifically, the problem in (9) is a non-cooperative game with respect to the payoff vector R. Therefore, it can be solved efficiently using interference game methods for convex non-cooperative N-player games. We solve the problem using the Gaussian Interference game model and consider it as a baseline to design the reward function of DRL scheme proposed in the next section. 

## 4. Deep RL-Assisted Resource Allocation

In this section, we first introduce the two fundamental RL algorithms, namely Q-learning and DQL, as representatives of the policy-based and value-based design techniques, respectively. Then, we define our proposed DQL-assisted resource allocation to address the optimization problem in the traditional method. Finally, we present the problem formulation and specific procedures of the proposed DQL-based power allocation model.

### 4.1. Basics of RL Algorithms

**Figure 2:**
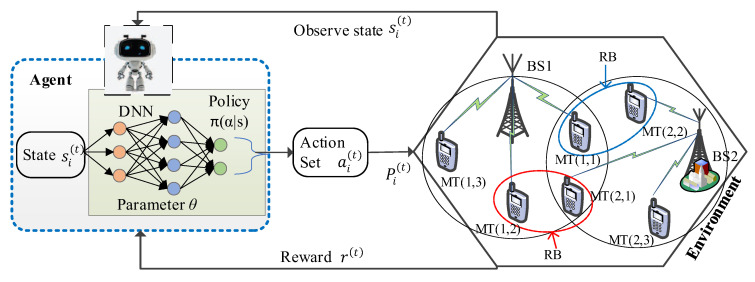
Reinforcement learning for multi-cell OFDMA systems.

The RL method addresses sequential decision-making by maximizing a cumulative reward function while interacting with the wireless environment, as shown in Figure 2. Assuming time series t, the agent observes the environment and receives a state and feedback regarding the observed states. For each time step t, the agent receives a state $s^{\left(t\right)}\in S$ from a state space and then selects a specific action $a^{\left(t\right)}\in A$, where S and A are a set of states and possible actions, respectively. After several executions, the agent receives a reward $r^{\left(t\right)}\in R$ and sends an action $a^{\left(t\right)}$ to the environment estimated by applying a certain policy π to the state $s^{\left(t\right)}$, where R:S×A→R denotes the reward function. The policy π is essentially undertaken by the agent in a given state and best possible action. The agent then follows a policy π(a,s) and maps the state $s^{\left(t\right)}$ to a probability distribution over a set of possible actions A. Once the agent executes action $a^{\left(t\right)}$, the environment changes its given state $s^{\left(t\right)}$ to a next state $s^{\left(t+1\right)}\in S$ in response to the agent’s action. This scheme is repeated until the agent reaches the ending state and restarts.

Q-learning (QL) is a basic form of RL algorithm aiming to evaluate the actions of the agent based on the current environment and maps the outputs in the form of rewards. The goal of QL algorithm is to find an optimal strategy $\pi_{*}$ that maximizes the future cumulative rewards starting from given state s, performing action a, while following policy π. Therefore, the future cumulative reward at time t is given by

(11) $$R_{t}=\sum_{k=0}^{\infty }\gamma^{k}r_{t+k}$$

where γ denotes the discount factor for long returns. The agent uses this discount factor to adjust the importance of securing rewards over time t. Multiple episodes are executed to train the QL algorithm, and the agent uses the ϵ-greedy policy to estimate the optimal strategy $\pi^{*}\left(s,a\right).$ We describe the Q-function $Q^{\pi }\left(s,a\right)$ for policy π, which is the expected reward beginning from current state s, selecting best action a, and thereafter following strategy π.

(12) $$Q^{\pi }\left(s,a\right)=E_{\pi }\left[R^{\left(t\right)}|s^{\left(t\right)}=s,\;a^{\left(t\right)}=a\right]$$

Similarly, the optimal strategy has an optimal Q-function, denoted as $Q^{*}$ and define as $Q^{*}\left(s,a\right)=\underset{\;\pi }{\max}Q^{\pi }\left(s,a\right)$ for s∈S and a∈A(s). On the other hand, $Q^{*}$ gives the maximum expected reward attainable by any strategy π for each possible state–action pair. So, the mathematically optimal Q-function can be expressed by the Bellman equation as:

(13) $$Q^{\pi }\left(s,a\right)=R\left(s,a\right)+\gamma \sum_{s^{\prime }}\left(P\left(s,a,s^{\prime }\right)\underset{\;a^{\prime }}{\max}Q^{\pi }\left(s^{\prime },a^{\prime }\right)\right)\;$$

where $s^{\prime }$ and $a^{\prime }$ denote the value of the new state and action, respectively. After state transition due to the actions taken and following a policy π, the agent stores all the optimal Q-values in a tabular form. The QL algorithm uses a lookup table, also known as a Q-table, to save the Q-values of the optimal function. The Q-table is a matrix in which the number of rows represents the states and the columns corresponding to the actions. Once this Q-table is constructed, at each time-step t, the agent chooses the best actions based on the ϵ-greedy strategy and ϵ is set to the value from the latest training step. Then, the agent executes a random action, and the action with the higher value is selected with probability 1 − ε to avoid getting stuck at non-optimal strategies. According to the ϵ-greedy policy, the agent either exploits the Q-table to obtain many rewards or explores the environment to select better action in the future. After obtaining a new experience due to the action taken, the QL algorithm updates the value of a Q-table based on the feedback learning agents. The QL update uses the following iterative approaches to train the QL algorithm:

(14) $$Q\left(s^{\left(t\right)},a^{\left(t\right)}\right)\leftarrow Q\left(s^{\left(t\right)},a^{\left(t\right)}\right)+\alpha \left[R^{\left(t+1\right)}+\gamma \underset{a^{\prime }}{\max}Q\left(s^{\left(t+1\right)},a^{\prime }\right)-Q\left(s^{\left(t\right)},a^{\left(t\right)}\right)\right]$$

Here, γ∈(0, 1] is a factor that determines the priority of future rewards compared to the current reward and lies in the [0:1] range. A value of γ=1 means that future rewards are more important than the current reward. Furthermore, α(0<α≤1)  is a learning rate, which denotes the proportion of newly learned data related to the given action value. When α=1, the agent can learn the policy, and the newly trained data are the only significant information. For each of the current Q-value $Q\left(s^{\left(t\right)},a^{\left(t\right)}\right)$, an estimate of the expected reward is tracked and an ϵ-greedy policy is selected based on these estimates. After that an arbitrary action is chosen with probability ϵ(0≤ϵ≤1) while the action with highest value is selected with probability 1−ϵ. Since the QL algorithm updates according to the Bellman equation, the testing or implementation phases can be executed through online temporal difference learning.

Traditional RL methods struggle to address real-world problems due to the inability to efficiently model high dimensional state space’s objective. The limitation of the QL algorithm is that it adopts the Q-table to store the Q-values. However, in many problems of practical scenarios, the Q-table will be hard to use when the state space of the problem is too large. Consequently, it is important to use function approximation to handle the large state-action spaces. In this paper, we focus on the deep Q-learning network (DQN), a combination of deep neural networks (DNN) and traditional QL. Based on the QL algorithm, DQN uses DNN to approximate the optimal action-value function $Q\left(s,a,\theta \right)\approx Q^{*}\left(s,a\right)$ on a discrete action space. Here, Q(s,a,θ) denotes the DQN and θ is the parameter of the neural network. Instead of using a table to store the Q-values, the DQN uses a replay memory D to store the transition tuples ($s^{\left(t\right)},a^{\left(t\right)},R^{\left(t+1\right)},s^{\left(t+1\right)}$) at each time period. The memory stores experiences to avoid correlation between input data in successive updates. The DQN is trained with a minibatch sampled randomly from the replay memory and updates the targets of that minibatch. The mean squared error (MSE) of the Bellman equation is minimized by the iterative update, which is used to train the Q-network. Hence, an experience replays to minimize the MSE, denoted as [30]:

(15) $$L\left(\theta \right)=\sum_{D}{\left[R^{\left(t+1\right)}+\gamma \underset{a^{\prime }}{\max}Q\left(s^{\prime },a^{\prime };\theta^{-}\right)-Q\left(s^{\left(t\right)},a^{\left(t\right)};\theta \right)\right]}^{2}$$

where $\theta^{-}$ denotes the parameter set of learning target action-value function, which is generated from the training DQN parameter θ periodically and fixed for successive updates. Note that the target of Problem (15) is to train the DQN function for a random mini-batch $D^{\left(t\right)}$ each time t, such that $R_{DQN}^{\left(t\right)}\left(s^{\prime },a^{\prime }\right)=r^{\prime }+\gamma \underset{a^{\prime }}{\max}\hat{Q}\left(s^{\prime },a^{\prime };\theta^{\left(t\right)}\right)$.

### 4.2. Proposed DQL-Based Approach

In this subsection, we present our proposed approach that can perform the downlink resource allocation on multi-cell OFDMA systems. We consider a single DQL-based algorithm in which the agent makes interaction with the environment in order to learn the optimal power allocation of the users. The wireless environment contains everything in the OFDMA downlink transmission strategies except the agent. In the DQL algorithm, the serving BS in different cells is used as an agent i and interacts with the environment by mapping the observed state and performing possible actions. The agent i transmits confidential information to each user using the transmit power $P_{i}$ and provides the required QoS guarantee. We denote the current state of agent i as $s_{i}\in S_{i}$, which is comprised of environment features that are applicable to the possible actions $a_{i}\in A_{i}$ of agent i. As represented in Figure 2, at the time step t, the two neighborhood BS sets are coupled together using the cross-coupling links $h_{ij}\left(k\right)$. Observe that, even though the strategy sets of the BSs are dependent on each other, they are also cross coupled in the same fashion, i.e., by the same QoS conditions $SINR_{i}\left(k\right)>\gamma_{o}$, for k=1,…,K. In this case, the interfering BS’s transmit power $P_{j}^{\left(t\right)}\left(k\right)\;h_{j\to i}^{\left(t\right)}$(k) will decrease due to its interference with neighboring BS-user pairs. Due to the cross coupling of BS-receiver pairs, our proposed DRL-based algorithm will be more complicated than the Nash equilibrium solution discussed in Section 2. Our aim is to measure the impact of each BS’s interference on its neighbors’ transmission systems, so we limit the exchange resources between neighboring BS-receiver pairs. Let the set of BSs whose $SINR_{i}$ at user i is greater than a given threshold $\gamma_{o}$ at each time step t be defined as

(16) $$I_{i}^{\left(t\right)}\left(k\right)=\left\{j\in K,\;j\neq i\backslash h_{j\to i}^{\left(t\right)}P_{j}^{\left(t\right)}\right\}>\gamma_{o}\sigma_{i}{}^{2}$$

where $I_{i}^{\left(t\right)}\left(k\right)$ denotes the set of “interferers” with respect to player i and $h_{j\to i}^{\left(t\right)}P_{j}^{\left(t\right)}$ represents the received interference power from BS i. Similarly, let the set of users whose SNR from BS i is also greater than the threshold value $\gamma_{o}$ at each time step t be represented as

(17) $$U_{i}^{\left(t\right)}\left(k\right)=\left\{j\in K,\;j\neq i\backslash h_{i\to j}^{\left(t\right)}P_{i}^{\left(t\right)}\right\}>\gamma_{o}\sigma_{i}{}^{2}$$

From the neighboring BS-user pairs perspective, $I_{i}^{\left(t\right)}\left(k\right)$ is the set of “interferers” while in contrast $U_{i}^{\left(t\right)}\left(k\right)$ represents the set of the “interfered” neighbors’ transmissions. Next, we consider the CSI the BS take over at each time step t. Here, we firstly assume that the BS i learns via direct channel response $h_{i,i}^{\left(t\right)}\left(k\right)$ in the same cell. Moreover, the BS i also learns the corresponding received $SINR_{i}$ at user i before the update of transmit power control, i.e., $\sum_{j\neq k,j\neq i}^{K}h_{j\to i}^{\left(t\right)}P_{j}^{\left(t\right)}+\sigma_{i}{}^{2}\left(k\right)$. Moreover, at each time step t, user i will inform interfering BS i of the received signal from its neighbors’ transmissions $j\in I_{i}^{\left(t\right)}\left(k\right)$, i.e., $h_{j\to i}^{\left(t\right)}P_{j}^{\left(t\right)}$. Note that these measurements can only be available at BS i at each time step t.

The objective of the training is to exploit a trained DQN to replay the experience in learning the power allocation strategy. We adopt DQL with experience replay to train our learning algorithm. The resource optimization scheme is performed in two phases, (1) in the learning or training stage, (2) in the testing or implementation phase. In the learning phase, the agent is in charge of the RB allocation and learns its best actions to achieve an optimal strategy by updating the RB allocated to each user. In the implementation phase, the agent evaluates each action and selects the action with the maximum award in the current environment that will be actually executed. In the following, we define the state and action spaces, and reward function for the proposed DQL approach.

#### 4.2.1. State Space 

The wireless environment is denoted by a set of variables analogous to the resource allocation issue, this variable set with all available values are referred to as the state space, and it is denoted by S. In our case, the state space comprises the resource allocation-related information of users and the channel gain from multiple cells, which follows a certain optimization strategy. More specifically, the state space S contains i states, i.e., $S=\left\{S_{1},S_{2},\ldots ,S_{i}\right\}$. As described above, the agent i constructs its state $S_{i}{}^{\left(t\right)}$ using information from the direct channel gain and cross coupling links. We denote by $S_{i}≜U_{i}$ the set of users connected to each base station and define $S_{i}≜\left\{U_{1},\ldots ,U_{i}\right\}$. At the initial interaction with the environment, agent i sends its interference signal by using cross coupled power $h_{j\to i}{}^{\left(t\right)}\left(k\right)p_{j}^{\left(t\right)}\left(k\right)$ from BS j at receiver i. In this case the agent i uses the weights of sum-rate $w_{i}$ to prioritize its interference signal. For direct information exchange, the agent i uses the direct gain $h_{i\to i}{}^{\left(t\right)}\left(k\right)$ at each time step t. For the coupling strategy, agent i uses the cross-coupling links $h_{j\to i}{}^{\left(t\right)}\left(k\right)$ at each time step t. For $S_{i}≜U_{i}$ user set in m cells and n frequency tones, we will have $m\times U_{i}\times \left(n+1\right)$ state size. Hence, the DQN agent uses S(m,n) vector along with $U_{i}$ of all users in a network as state and then takes action.

#### 4.2.2. Action Space

The action space is the set of all available actions the agent takes from the current state, denoted as $a^{\left(t\right)}\in A$. In this paper, we assumed that the agent is in charge of the RB allocation and the DQN algorithm comes down to optimizing transmit power control for each user in a multi-cell network. More specifically, the total number of possible actions relies on the number of power levels the BS can distribute to the corresponding receivers. Different actions in the set of all available action spaces denote different power controls that the BS can schedule to mobile users in the multi-cell system. Even though the transmission system mostly adopts continuous power values for minimizing complexity, in this paper, we use discrete power levels between 0 and $P_{max}$. We denote by $A_{m}$ the (non-empty) set of action spaces assigned to cell m and defined $a_{m}\in A_{m}$ is the random selected action for cell m. For each cell, we have n number of actions, and then the number of actions for M number of cells will be M×n. Each action $a_{m}\in A_{m}$ corresponds to the power levels we are using. The possible emitting power is quantized exponentially in |A|−1 levels along with a zero-power level, which indicates that there is no signal transmission. For each cell m, we assume that the action space has $\left|A_{m}\right|>1$ discrete power levels. Therefore, the action space can be expressed as

(18) $$A_{m}=0,\frac{P_{max}}{\left|A_{m}\right|-1},\;\frac{2P_{max}}{\left|A_{m}\right|-1},\;\ldots ,P_{max}$$

Here, if there is no other described information regarding the wireless environment, the DQN agent performs action formation $a_{m}=\arg\underset{a}{\max}Q^{k}\left(s_{t},a_{t};\theta \right)$ with probability ϵ. 

#### 4.2.3. Reward Function

Reinforcement learning (RL) aims to solve problems that are hard to optimize using the traditional framework. This is typically tackled by considering the overall goal of the problem and designing the reward signal that correlates with the ultimate goal. In our proposed algorithm, the target function is defined to evaluate the agent’s actions and outputs the result in a positive reward or penalty. Here, the maximum throughput of the system corresponds to a positive reward. Moreover, a higher received signal for each user benefits to improve the system throughput and award. On the other hand, to ensure the QoS requirement, the achievable rate of each user that satisfies the desired threshold aids to achieve a good reward, while the achievable rate that does not fulfil the required outcomes is given a negative reward or penalty. 

Taking into account the above points, we explain the reward function as how the strategy $P_{i}^{\left(t\right)}$ affects the sum rate at time step t. As described in (6), we set the design objective to maximize the sum throughput while ensuring the QoS conditions for each user. At each time step t, for all agent i∈K, the training network computes the achievable rate of each user i without the coupling interference from BS i. Accordingly, we can describe the network trainer function for all users $U_{i}$ at time period t as follows:

(19) $$R_{\tilde{i}}^{\left(t\right)}\left(k\right)=\log_{2}\left(1+\frac{P_{i}^{\left(t\right)}\left(k\right){\left|h_{i\to i}^{\left(t\right)}\left(k\right)\right|}^{2}}{\sum_{j\neq k}^{K}h_{j\to i}^{\left(t\right)}P_{j}^{\left(t\right)}+\sigma_{i}{}^{2}\left(k\right)}\right)$$

Furthermore, the Q-network trainer estimates the cross coupling plus noise term (i.e., $\sum_{j\neq k,j\neq i}^{K}h_{j\to i}^{\left(t\right)}P_{j}^{\left(t\right)}+\sigma_{i}{}^{2}\left(k\right)$) in (18) by simply subtracting $h_{i\to i}^{\left(t\right)}P_{i}^{\left(t\right)}$ from the total SINR power term in time step t. Thus, the reward function in the absence of QoS at time step t is given by

(20) $$r_{i}^{t}=w_{i}^{t}\left(R_{i}{}^{\left(t\right)},\ldots ,R_{N}{}^{\left(t\right)}\right)$$

In addition, we also consider the reward signal design intending to optimize the overall capacity of the entire system under the relevant QoS conditions. The spectral efficiency of each BS i is its rate $R_{i}^{\left(t\right)}\left(k\right)$, and each BS has to satisfy the constraints of power budget and QoS requirements. As mentioned in Problem (16), since the BS $i\in I_{i}^{\left(t\right)}\left(k\right)$, its interference channel K in time step t, i.e., $h_{j\to i}^{\left(t\right)}P_{j}^{\left(t\right)}>\gamma_{o}\sigma_{i}{}^{2}$ is perfectly measurable by user each user i and can be generated to the training DQN. At each time step t, we consider each BS’s interference on its neighbors’ transmissions that an interfering BS $i\in I_{i}^{\left(t\right)}\left(k\right)$ causes to its neighboring BS-user pairs using interference pricing. Accordingly, the impact of each BS’s interference on its neighbors’ transmissions can be given by [52] U

(21) $$\pi_{\tilde{i}}^{\left(t\right)}=w_{i}^{t}\left(R_{\tilde{i}}^{\left(t\right)}\left(k\right)-R_{i}{}^{\left(t\right)}\right)$$

In this paper, we assume that $w_{i}=1$ to maximize the sum throughput. To ensure that the QoS constraint is satisfied, the condition $SINR_{i}\left(k\right)>\gamma_{o}\left(k\right)$ is checked by agent i∈K in the reward estimation. If the QoS does not meet this requirement, the algorithm simply declares the power $P_{i}^{\left(t\right)}$ selection of that user as wrong and sets the reward to zero. Therefore, the QoS constrained reward design at time step t can be formulated as

(22) $$R_{i}^{t}=\{\begin{array}{c} \left(R_{i}{}^{\left(t\right)}-\sum_{i\in U_{i}^{\left(t\right)}\left(k\right)}\pi_{\tilde{i}}^{\left(t\right)}\right),\;if\;\frac{P_{i}\left(k\right){\left|h_{ii}\left(k\right)\right|}^{2}}{\sum_{j\neq k}^{K}P_{j}\left(k\right){\left|h_{ij}\left(k\right)\right|}^{2}+\sigma_{i}{}^{2}\left(k\right)}>\gamma_{o}\left(k\right)\;\;\\ 0,\;\;\;\;\;\;\;\;\;\;\;otherwise\;\;\;\;\;\;\;\;\;\;\;\;\;\;\;\;\;\;\;\;\;\;\;\;\;\;\;\;\;\;\;\;\;\;\;\;\;\;\;\;\;\;\;\;\;\;\;\;\;\; \end{array}$$

According to the problems mentioned above, we can find that the reward of agent i is composed of three main components: (i) the direct contribution to the original problem in (6) by the same QoS constraints, (ii) the reward due to the game player satisfying the QoS and power constraints, and (iii) the penalty due to the cross-coupling interference and each player which does not meet the requirement to QoS target. In addition, when the peak transmission power $P_{i}^{\left(t\right)}$ at the given time period t equal to $P_{i}^{max}$, the positive contribution and penalty will be maximized, whereas being silent gains zero reward.

### 4.3. DQL-Algorithm Description

The goal of the DQN agent is to maximize the total weighted sum throughput, as shown in (6), and assumes the throughput of each user can be evaluated based on the SIR requirements. The learning process begins from an initial state $S_{i}{}^{\left(t\right)}$ and continues as long as the throughput increases by executing the possible actions. The agent’s possible actions $a_{i}{}^{\left(t\right)}$ to update the resource allocation for different users can be chosen by the following approaches. (i) allocate a free RB with more SIR to different users in the same cell, (ii) update the allocated resource to the user with the worst SIR in the same cell, (iii) For a certain RB, cluster the users with the best SIR in the neighboring cell with user with the worst SIR in the same cell. Note that the third strategy is used to avoid assigning the same RB to users in neighboring cells that are positioned near to the edge of each cell. Once all possible actions of the agent are selected, the agent gives more emphasis on maximizing the overall capacity of the entire system. 

#### 4.3.1. Training the DQL Algorithm

The objective of the training model is to utilize a trained DQN to reply to the accumulated experience in training the power allocation strategy. The overall procedure for training the DQL algorithm is presented in Algorithm 1. We first define the essential parameters of the Q-network, such as discount factor, learning rate, number of layers, and activation functions. Here, we directly assume that the input and output layer sizes are the same as the state space and the total number of action sizes, respectively. At the output layer, the individual slot provides the approximate of training DQN with current state input and the possible action output. In the initialization phase, we first define the user’s channel information, replay memory D, the action-value function Q and the learning target DQN $\hat{Q}$. In particular, the DQN with experience replay is used to train the DQN function Q [30]. The DQN takes the current observable state $s^{\left(t\right)}$ as input and outputs the agent’s possible action-value. Multiple episodes are executed to train the DQN function and accumulate experiences with its connection with the environment. Since we utilize a single DQN agent scenario, the state transition of the environment relies upon the agent’s possible actions.
**Algorithm 1** DQL-Based Resource Allocation 1: Initialize the current environment E (all available CSI) 2: $B_{U_{i}}\leftarrow B$% Initialize usable sub-bands for all users 3: Initialize replay memory D to capacity M 4. Initialize the state space S and action space A 5: Initialize train DQN Q and target DQN $\hat{Q}$ with weights θ & $\theta^{-}$, respectively. 6: $\left\{BS_{1},BS_{2},BS_{1},\ldots ,BS_{k}\right\}\leftarrow BS$, where $BS\in B_{U_{i}}$ 7: **for** each episode do 8:    Allocate a free RB with higher SIR for $U_{i}$ in each cell. 9:    **for** t=1,…,∞ **do**
10:     $SINR_{i}$  % calculate the SINR for $U_{i}$ in the network 11:     Use the throughput of each user as state space $S_{i}{}^{\left(t\right)}$
12:     **for** i=1,…,K **do** 13:       Select a random action $a_{k}^{\left(t\right)}$ from A with probability ϵ for user k. 14:       Otherwise choose $a_{k}=\arg\underset{a}{\max}Q^{k}\left(s_{i}{}^{\left(t\right)},a_{i}{}^{\left(t\right)}\right)$
15:     **end for** 16:     Execute action $a_{k}=\left[a_{1},a_{2},\ldots ,a_{k}\right]$ and observe reward $r_{t}$% calculate $w_{i}^{t}R_{i}{}^{\left(t\right)}$ the throughput for entire system. 17:     State transition happens $s_{t+1}$ & move from $s_{t}\;\mathrm{to}$ $s_{t+1}$
18:     Store all transition tuples $\left(s^{\left(t\right)},a^{\left(t\right)},R^{\left(t+1\right)},s^{\left(t+1\right)}\right)$ in D. 19:     Sample mini-batch from D and train it at each time t, $D^{\left(t\right)}$. 20:     Optimize the loss function between learning target $\hat{Q}$ and train Q-function using tools of stochastic gradient descent algorithm, (15). 21:     Update the target DQN $\hat{Q}$ to be equal to train DQN Q. 22:   **end for**
23: **end for**

After the transition of the environment due to the change of CSI and the actions taken, the agent stores all the transition tuple $\left(s^{\left(t\right)},a^{\left(t\right)},R^{\left(t+1\right)},s^{\left(t+1\right)}\right)$ in a replay memory. At each step, a mini-batch of accumulated experience is sampled randomly from memory. The mini batch is adopted to train the DQN and a stochastic gradient-descent step is used to update the action-value DQN parameters. The objective is to minimize the sum-squared error based on (15) at each time step t. The target DQN function $\hat{Q}$ is initialized by duplicating the parameters of the training DQN function Q and after a certain amount of time the two DQNs are updated to clone the parameters of the training DQN. The process of training the DQN is repeated until the parameters converge. 

#### 4.3.2. Testing the DQL Model

In the testing phase, at time step t, the agent or the network trainer takes action $a_{i}{}^{\left(t\right)}$ and receives the experiences of available channel information $s_{i}{}^{\left(t\right)}$ based on the current decision policy. For testing the DQL Model, ϵ is set to the value from the very last training step. Since the agent is working to maximize the original problem in (6) with relevant reward signal design has been discussed in Section 4.2.3, it can benefit from this experience. After that the agent evaluates the action with the maximum value at the training DQN output. Once the agent chooses the action corresponding to transmit power, it starts to adjust the RB allocation to maximize the overall capacity of the entire system. Each BS then learns its optimal power allocation strategy, which runs the DQL model. Note that the training procedure presented in Algorithm 1 can be performed offline because it is computationally expensive for a large number of channel conditions. On the other hand, for computationally inexpensive scenarios, the testing phase can be performed online for the actual deployment of the network.

## 5. Numerical Simulations

In this section, we intend to present the performance evaluation of the proposed DQL-based power allocation scheme via numerical simulations. We consider a downlink OFDMA transmission with random distribution of BSs and mobile devices over multiple channels in multi-cell network as shown in Figure 2. It has to be noted that the path loss and channel coefficients are modeled using the standards of independent and identically distributed complex Gaussian noise, while the power of the additive noise at each receiver has been normalized. According to [53], the path loss related to distance is given by $128.1+37.6\;\log_{10}d$ dB, where d is the distance between the serving BS and each receiver in kms. The maximum transmit power budget for BS over multiple channels is given as 33 dBm, which is reusable in multi-cells. For the downlink OFDMA, all users share a bandwidth B and each user occupies of B/N, where B is given as 10 MHz and N is the number of receivers. The capacity requirement for each receiver is 1b/s/Hz. The additive noise power at each user is −114 dBm and the noise PSD is set to −174 Bm.

We next define the training parameters for our DQN model. Determination of these hyperparameter values becomes more challenging in DL-based resource allocation [42]. In this work, we do not over-parameterize the structure of a neural network. Our goal is to accelerate the learning process, so we adopt a small architecture of deep neural networks for training purposes. To train a DQN, we use a deep neural network with three hidden layers, including 200, 100, and 40 neurons, where both the input and output layers are taken as 1. We also use the ReLU, f(x)=max(0,x) as an activation function for the hidden layers. We assume that the F RBs are equally distributed between N channels, which exhibiting the same bandwidth B. Each linear unit has F number of RB allocation that may be grouped in N channels. ReLU is used to avoid the vanishing gradient problem and allows the DQL model to learn faster and perform better [54]. We also normalize the input layer size of the Q-network with some parameters relying on the maximum total power constraint, inter-cell path loss, etc., to optimize the performance metrics. Furthermore, we use the RMSProp optimizer method with learning rate of 0.001 for updating the weights of the DQN function. Given the value of discount factor γ=0.5 and updating the DQN over 3000 episodes. However, as the value of discount factor γ increases, the outcome of DQL keeps improving for most of its applications [35]. We also apply the ϵ-greedy algorithm to facilitate the training of DQN network. Herein, the DQN agent takes action randomly with probability ϵ and can control the search algorithm by adjusting the value of ϵ.

Performance evaluation in terms of sum throughput maximization, power consumption and spectral capacity will be carried out to compare the proposed method with several methods in the simulations. The proposed method uses several benchmarks, such as a ‘WMMSE’ method developed in [5] and an ‘ideal WMMSE’ with instantaneous full CSI. Furthermore, we also use the downlink OFDMA with random power allocation method (‘OFDMA random’) and ‘full power allocation’ scenarios. In the case of ‘OFDMA random’, the DQL agent will choose its transmitted power for multiple channels at random between 0 and $P_{max}$ while in the ‘full power allocation’, the agent will use the maximum transmit power for all channels.

**Figure 3:**
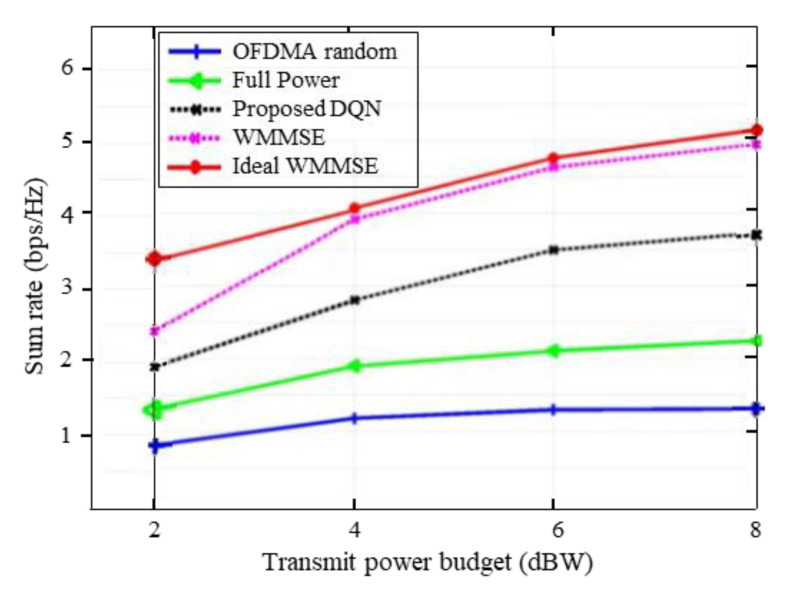
Sum-rate vs. transmit power budget for different schemes.

Figure 3 illustrates the sum throughput performance of different power allocation methods in a 5-user scenario with QoS threshold $\gamma_{o}=3$. It can be seen from the figure that the sum throughput of the proposed DQN method and the state-of-the-art power allocation algorithms increases with an increasing of transmit power, which contributes to improving the achievable weighted sum rate performance. The WMMSE approach in [5] and ideal WMMSE with perfect CSI provide better sum-rate performance due to higher available sub-bands for each cell network while these methods do not maintain the QoS constraints. The proposed DQN scheme however considers the QoS constraint and achieves a higher sum rate than the full-power and random power allocation methods. Furthermore, the protection of cell-edge users in neighboring transmissions against severe cross-coupling interference in the proposed scheme adds to its performance. As expected, the full-power and random OFDMA allocations tend to display lower sum throughput performance compared to the proposed DQN and WMMSE algorithms. For the random OFDMA-based system, since each user uses the $P_{max}$, the throughput obtained does not change with the sum-rate requirements.

**Figure 4:**
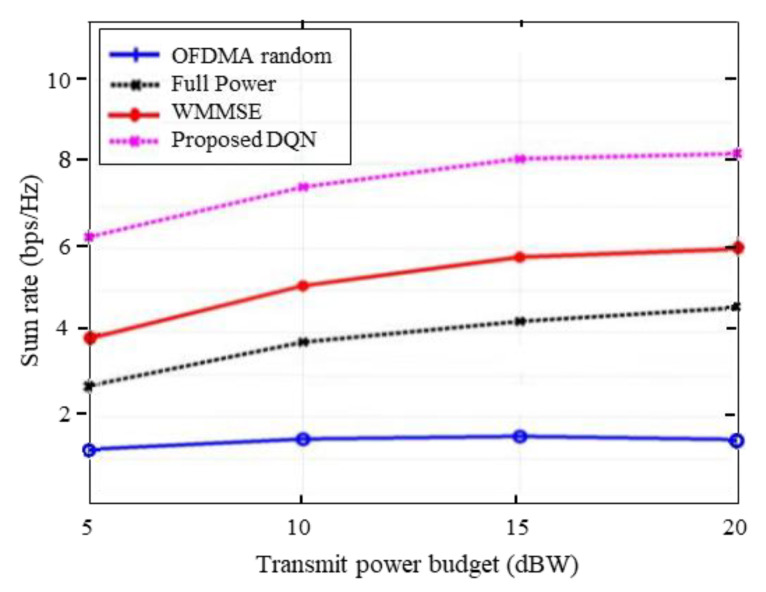
Sum rate vs. power budget ignoring QoS.

To further elaborate on the performance of our proposed method, we plot the average sum throughput versus the transmit power budget with the absence of a QoS constraint in Figure 4. It can be seen from the Figure that the proposed DQN approach achieves a higher sum throughput than the WMMSE and random OFDMA methods. This setting has been discussed with the reward design in Problem (20). When we ignore the QoS constraint, the reward signal is designed to optimize the utility function of each BS i and capable of obtaining better sum-rate performance. Indeed, the proposed scheme is aiming to maximize the overall capacity of the entire system under the QoS conditions and has also achieved the better result. From Figure 3, it is evident that with the increase of the transmit power budget, the proposed DQN approach is capable of obtaining a better result with distributed execution while satisfying the QoS requirement.

**Figure 5:**
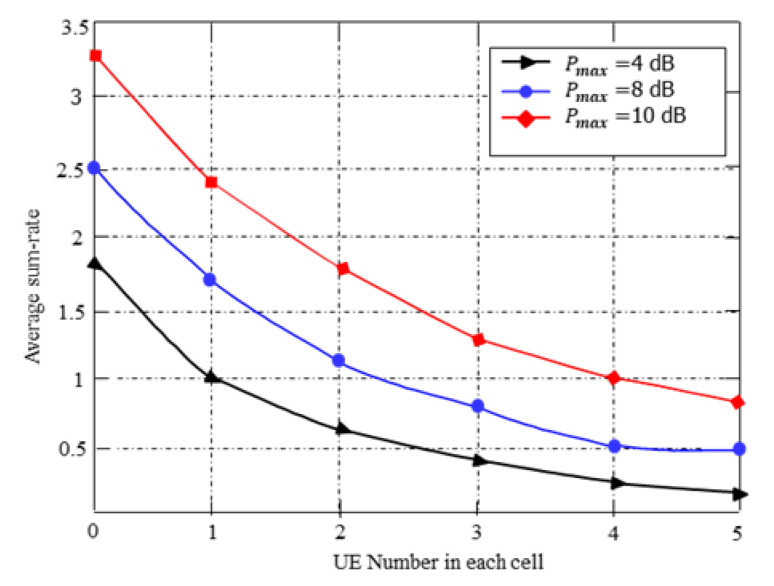
The average sum rate vs. the number of user pairs.

Figure 5 shows the relationship between the average sum rate and the number of users with different power budgets. From the figure, it is found that the average sum rate slowly decreases with the cell user number increasing for varying transmit power budgets. As expected, when the user’s number increases, the average sum throughput performance of the proposed approach decreases gradually because, in such situations, additional interference will generate when the cell users become large. However, as evident from Figure 5, increasing transmit power budget of each user leads to a compromise.

### Computational Complexity Analysis

In order to solve the original NP-hard problem in (6), we further unified it under the Gaussian interference game framework in (9). We realize the potential of applying the DRL techniques on various resource-limited networks as proof of the optimization techniques. Then, a DRL-based solution with experience replay is developed to reduce computational complexity. The performance of the DQL-based power allocation model is evaluated for several training scenarios, including increasing cell user demands, varying transmit power budgets. We have run the learning process an average of 9 to 10 randomly initialized experiments. The computational complexity of the learning process is inherently addressed using the two separate DQN functions (i.e., the train DQN and learning target DQN with parameters set θ). For comparison purposes, we use the sum rate performance metrics for the optimization solution achieved through different power allocation methods. As can be observed from the numerical simulations, it is evident that the proposed DQL approach performs better than other power allocation methods.

As expected, the complexity of the DRL algorithm mainly relies on the wireless network sizes. According to Figure 5, it is evident that with the increase in the number of destination receivers, the average sum throughput performance of the proposed DRL-based approach decreases gradually. This is because, with the increase of cell users, the wireless network size increases while the state-action space also increases accordingly. As a result, the learning algorithm can figure out more exploration to estimate the optimal action-value functions. This is why the sum-rate performance of the DQN model decreases gradually with the increase of cell users.

## 6. Conclusions

In this paper, we have studied a distributed wireless interference system for OFDMA networks. In particular, we proposed a DRL-based resource optimization scheme in a downlink multi-cell OFDMA system and investigated the weighted sum throughput maximization problem. To maximize the sum rate, the objective function was first formulated using a non-cooperative game model. Then, we developed a DRL-based scheme that interactively learns the resource allocation over multiple interference channels while satisfying the QoS requirements for each user. We utilized a DQN with experience replay to understand the transmission strategies for the proposed DQL scheme. We have evaluated the performance of the proposed distributive optimization approach with other power allocation methods, such as random OFDMA, WMMSE, and instantaneous maximum power allocation schemes. Our theoretical investigation and numerical simulations demonstrate that the proposed optimization method can improve the sum rate performance while guaranteeing each user’s throughput.

## Data Availability

Not applicable.

## Abbreviations

5G	The Fifth Generation Mobile Networks
AN	Artificial Noise
AWGN	Additive White Gaussian Noise
CSI	Channel state information
DC	Difference of convex function
DL	Deep learning
DNN	Deep neural network
DQL	Deep Q-learning
DQN	Deep Q-learning network
DRL	Deep reinforcement learning
FDM	Frequency division multiplexing
FP	Fractional programming
GIG	Gaussian interference game
GNEP	Generalized Nash equilibrium problem
KPI	Key performance indicator
ML	Machine learning
MSE	Mean squared error
NP-hard	Non-polynomial hard
OFDMA	Orthogonal frequency-division multiple access
PSD	Power spectral density
QoS	Quality of service
RB	Resource block
ReLU	Rectified linear activation unit
RL	Reinforcement learning
SIPNR	Signal-to-interference-plus-noise-ratio
WMMSE	Weighted-minimum mean squared error
